# Successful sofosbuvir treatment with ribavirin dose reduction for chronic hepatitis C virus genotype 2 infection in a patient with ulcerative colitis: a case report

**DOI:** 10.1186/s12876-016-0480-x

**Published:** 2016-07-11

**Authors:** Yuki Ohta, Tatsuo Kanda, Tatsuro Katsuno, Shin Yasui, Yuki Haga, Reina Sasaki, Masato Nakamura, Shuang Wu, Shingo Nakamoto, Makoto Arai, Osamu Yokosuka

**Affiliations:** Department of Gastroenterology and Nephrology, Chiba University, Graduate School of Medicine, 1-8-1 Inohana, Chuo-ku, Chiba 260-8670 Japan; Department of Molecular Virology, Chiba University, Graduate School of Medicine, 1-8-1 Inohana, Chuo-ku, Chiba 260-8670 Japan

**Keywords:** Case report, Direct-acting antiviral (DAA), Hepatitis C virus (HCV), Interferon-free, Ribavirin, Ulcerative colitis

## Abstract

**Background:**

Ulcerative colitis is a lifelong, immunologically mediated disease. Direct-acting antivirals (DAAs) are now available for the treatment of chronic hepatitis C virus (HCV) infection. An interferon-free regimen appears useful, safe and effective for many patients for whom interferon-based treatment is contraindicated.

**Case presentation:**

We studied a 56-year-old treatment-naïve Japanese man with chronic HCV genotype 2b infection who had ulcerative colitis. This patient was treated with sofosbuvir and ribavirin for 12 weeks. During treatment, diarrhoea and bloody faeces were frequent. After ribavirin was reduced to 400 mg daily, these symptoms decreased. Finally, the patient achieved a sustained virologic response 12 weeks after the stoppage of the treatment.

**Conclusion:**

Clinicians should pay careful attention to the ribavirin dose in the treatment of certain HCV patients with inflammatory bowel disease who are receiving sofosbuvir plus ribavirin.

## Background

Inflammatory bowel disease (IBD), which comprises ulcerative colitis and Crohn’s disease, is a chronic immunologically mediated disease [1]. There are high-prevalence populations of IBD in North America and Europe [1]. In India and Japan, the incidence is increasing [1]. Ulcerative colitis is a lifelong, immunologically mediated disease and results from the inappropriate activation of the mucosal immune system by intestinal luminal antigens [2], although the progress in treatment of ulcerative colitis has been observed [2, 3].

The prevalences of hepatitis B virus (HBV) and hepatitis C virus (HCV) in patients with IBD are similar to those in the general population [4, 5], although these data are controversial [6, 7]. Because the prevalences of HBV and HCV are higher in Asian countries, including Japan, than those in non-Asian countries [8, 9], the management of these infectious diseases is still important in patients with IBD.

Interferon-α, which was previously the most common treatment for HCV, is a proinflammatory cytokine and can provoke a relapse of ulcerative colitis [10]. The synthetic guanosine analogue ribavirin (1-beta-D-ribofuranosyl-1,2,4-triazole-3-carboxamide) is also used for HCV eradication [11, 12]. The exacerbation of ulcerative colitis has also been reported during and/or after combination therapy with peginterferon plus ribavirin for chronic hepatitis C [13–15].

Recent advances in the treatment of patients infected with HCV make it possible to eradicate this virus with interferon-free regimens. Sofosbuvir is a potent nucleotide inhibitor for HCV RNA-dependent polymerase [16]. The combination of sofosbuvir and ribavirin led to higher sustained virologic response (SVR) rates in HCV genotype 2-infected individuals [16]. We report an ulcerative colitis-patient who was chronically infected with HCV genotype 2 and was successfully treated with sofosbuvir plus dose reduction of ribavirin for 12 weeks.

## Case presentation

A 56-year-old man with a 31-year history of ulcerative colitis was diagnosed with HCV infection at age 46. The patient received a blood transfusion at age 4 when he had surgery for his jaw and denied other risk factors for HCV infection, including tattoos or intravenous drug use. The patient drank alcohol (21 g daily) for 20 years; had a medical history of hypertension, IgA nephropathy, type 2 diabetes mellitus and dilated cardiomyopathy; and took several medications for these diseases. His ulcerative colitis was relatively well-controlled with oral sarazopyridine (4500 mg daily) and a sarazopyridine suppository (300 mg daily). The HCV genotype was 2b, and the patient was interferon treatment-naïve because he had ulcerative colitis, an autoimmune disease.

The patient’s height and body weight were 172 cm and 88.3 kg, respectively, and his body temperature was 35.2 °C. His laboratory findings before treatment are shown in Table 1. Abdominal ultrasound findings showed no masses in the liver and no ascites. Liver stiffness as measured by transient elastography was 7.6 kPa, indicating the absence of cirrhosis. Although he had chronic significant amount of alcohol intake, the ultrasound findings did not show the fatty change of the liver. He wanted to be treated for his chronic hepatitis C.

**Table 1:** Laboratory findings before treatment

Item	Value	Item	Value	Item	Value
AST	51 IU/L	BUN	16 mg/dL	HBsAg	negative
ALT	66 IU/L	Creatinine	0.68 mg/dL	Anti-HCV	positive
LDH	256 IU/L	UA	4.3 mg/dL	HCV RNA	6.4 logIU/mL
ALP	96 IU/L	Na	136 mEq/L	HCV genotype	2b
γ-GTP	88 IU/L	K	3.9 mEq/L	Anti-HIV	negative
T. Bil	1.2 mg/dL	Cl	104 mEq/L	Hyaluronic acid	37 ng/mL
TP	7.1 g/dL	WBC	5900/μL	AFP	7.4 ng/mL
Albumin	3.9 g/dL	RBC	477 × 10^4^/μL	PIVKA-II	32 mAU/mL
Amylase	83 IU/L	Hemoglobin	14.4 g/dL	NH_3_	58 μg/dL
CPK	102 IU/L	Haematocrit	40.7 %	HbA1c	7.8 %
T.CHO	115 mg/dL	Platelets	15.6 × 10^4^/μL	CRP	0.1 mg/dL
TG	103 mg/dL	PT	100 %		

*AFP* α-Fetoprotein, *ALP* alkaline phosphatase, *BUN* blood urea nitrogen, *CPK* creatine phosphokinase, *CRP* C-reactive protein, *HbA1c* haemoglobin A1c, *LDH* lactate dehydrogenase, *PIVKA-II* protein induced by vitamin K absence or antagonists-II, *PT* prothrombin time, *RBC* red blood cell count, *T.Bil* total bilirubin, *T.CHO* total cholesterol, *TG* triglyceride, *TP* total protein, *UA* uric acid, *WBC* white blood cell count

**Fig. 1:**
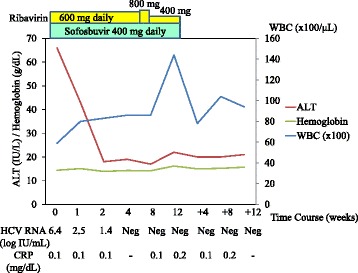
Clinical course of the patient. *ALT* alanine transaminase, *WBC* white blood cells, *w* weeks, *Neg* negative

The patient was treated with sofosbuvir at 400 mg daily and ribavirin at 600 mg daily. One week after the initiation of this treatment, the patient felt general malaise without any adverse events [white blood cell count (WBC), 7900/μL; hemoglobin, 14.7 g/dL; C-reactive protein (CRP) 0.0 mg/dL; AST, 35 IU/L; and ALT, 49 IU/L]. By week 3, the patient was having up to 10 loose bowel movements per day, with small amounts of blood. As the patient’s HCV RNA became negative and he improved to having 5 loose bowel movements per day by week 4 [WBC, 8600/μL; hemoglobin, 14.3 g/dL; AST, 19 IU/L; and ALT, 19 IU/L], the dose of ribavirin was increased to 800 mg daily. By week 7, however, the patient was having up to 20 loose bowel movements per day, with small amounts of blood, and the dose of ribavirin was decreased to 400 mg daily [WBC, 8200/μL; hemoglobin, 14.7 g/dL; CRP 0.1 mg/dL; AST, 19 IU/L; and ALT, 17 IU/L]. By week 8, the patient improved to having 10 loose bowel movements per day [WBC, 8600/μL; hemoglobin, 14.2 g/dL; CRP 0.1 mg/dL; AST, 20 IU/L; and ALT, 17 IU/L], and by week 11, his diarrhoea had resolved. Finally, the patient was treated with sofosbuvir plus ribavirin for 12 weeks. By week 12 after the initiation of this treatment, the patient’s HCV RNA was negative, and he had achieved a SVR 12 weeks (SVR12) after the stoppage of treatment [WBC, 14400/μL; hemoglobin, 16.2 g/dL; CRP 0.2 mg/dL; AST, 22 IU/L; and ALT, 22 IU/L] (Fig. 1). Three weeks post-treatment, an endoscopic examination of the colon-rectum confirmed that the mucosa was oedematous from the colon transversum to the rectum, although mucosal vascular permeability was reduced from the sigmoid colon to the rectum. The patient did not complain of abdominal pain or fever during treatment.

## Discussion

In the present report, we present a 56-year-old HCV genotype 2-infected patient who was diagnosed with ulcerative colitis and achieved a SVR12 after combination treatment with sofosbuvir plus ribavirin for 12 weeks. In Japanese multicentre, open-label phase 3 trial, HCV genotype 2-infected patients received 12 weeks of treatment with 400 mg of sofosbuvir, administered orally once daily, and ribavirin, administered orally twice daily, with doses determined according to body weight (1000 mg daily in patients with a body weight of >80 kg) [17]. But initial dose of the present case was 600 mg daily because we are afraid of adverse events of ribavirin. During treatment, the patient’s diarrhoea worsened, and the reduction of ribavirin with no reduction of sofosbuvir led to improvement in this symptom and the completion of therapy.

The mechanism of the inhibition of HCV replication by ribavirin is as follows: (1) ribavirin induces mutagenesis in HCV RNA; (2) ribavirin inhibits HCV RNA-dependent polymerase; (3) ribavirin inhibits the inosine monophosphate dehydrogenase enzyme and reduces intracellular guanosine pools, which are essential for HCV replication; and (4) ribavirin stimulates the T helper 1 (Th1) antiviral response, which leads to HCV eradication [8, 11, 12]. Ulcerative colitis is vaguely associated with abnormal Th2 immunity [18]. Although we do not know the exact effects of ribavirin on mucosal immunity, ribavirin worsened the symptoms of the present case with ulcerative colitis.

Although 12-week-treatment with sofosbuvir and ribavirin could lead to diarrhoea in 9 % of HCV genotype 2/3-infected patients [16], of interest, 12-week-treatment with sofosbuvir and ledipasvir could lead to diarrhoea in only 4 % of HCV genotype 1-infected patients [19]. We could not completely rule out the possibility that the combination of sofosbuvir plus ribavirin might be responsible, compared the only ribavirin.

One year before treatment, an endoscopic examination of the colon-rectum demonstrated that mucosal vascular permeability was reduced from the sigmoid colon to the rectum with marked mucus exudates. We could not completely rule out the possibility that this patient had baseline active mucosal disease but asymptomatic and then developed diarrhoea due to the sofosbuvir and ribavirin treatment. Although one important differential diagnosis of flare of ulcerative colitis is infection, as this patient did not have a high fever, we did not perform stool culture or use any antibiotics other than sarazopyridine. So we excluded bacterial infection in this patient.

HCV infection is a current leading cause of HCC in Japan and the United States [12]. This patient should be followed up for the occurrence of HCC [12]. Coexistence of IBD and chronic liver diseases, including chronic hepatitis C, leads to higher mortality rates than IBD alone [20]. Interferon-free therapy with or without ribavirin could increase treatment efficacy and shorten treatment duration compared with previous standards of care, such as peginterferon plus ribavirin treatment. However, the clinician should pay special attention to the use of ribavirin in the management of HCV-infected patients with IBD.

## Conclusion

We studied a HCV genotype 2b-infected patient with an ulcerative colitis exacerbation during sofosbuvir plus ribavirin treatment. The reduction of ribavirin improved this symptom, and the patient finally achieved a SVR12. Clinicians should pay careful attention to the ribavirin dose in the treatment of certain HCV patients with inflammatory bowel diseases, such as ulcerative colitis, in sofosbuvir plus ribavirin treatment.

## Abbreviations

HBV, hepatitis B virus; HCV, hepatitis C virus; IBD, inflammatory bowel disease; SVR, sustained virologic response; SVR12, SVR at 12 weeks; Th, T helper
